# Microbial biotechnology and circular economy in wastewater treatment

**DOI:** 10.1111/1751-7915.12821

**Published:** 2017-08-22

**Authors:** Per Halkjær Nielsen

**Affiliations:** ^1^ Department of Chemistry and Bioscience Center for Microbial Communities Aalborg University Fredrik Bajers Vej 7H Aalborg Denmark

## Abstract

Microbial biotechnology is essential for the development of circular economy in wastewater treatment by integrating energy production and resource recovery into the production of clean water. A comprehensive knowledge about identity, physiology, ecology, and population dynamics of process‐critical microorganisms will improve process stability, reduce CO2 footprints, optimize recovery and bioenergy production, and help finding new approaches and solutions. Examples of research needs and perspectives are provided, demonstrating the great importance of microbial biotechnology.

Wastewater treatment is becoming part of the circular sustainability movement by integrating energy production and resource recovery into the production of clean water. Microbial biotechnology is essential to this development given microbial communities can carry out key processes and combine these in different ways, i.e. removal or reuse of carbon (C), nitrogen (N), phosphorus (P), and micropollutants, and production of bioenergy and high‐value products. The future challenges are to optimize existing systems, e.g. minimize the footprint, reduce the requirement for chemical addition, lower energy inputs, improve process stability, to optimize recovery and bioenergy production and to find new approaches and solutions. This will help to reach the sustainable development goal 6: ‘Ensure availability and sustainable management of water and sanitation for all’ and, to some extent, also goal 7: ‘Ensure access to affordable, reliable, sustainable, and modern energy for all’.

## State of the art

**Figure 1:**
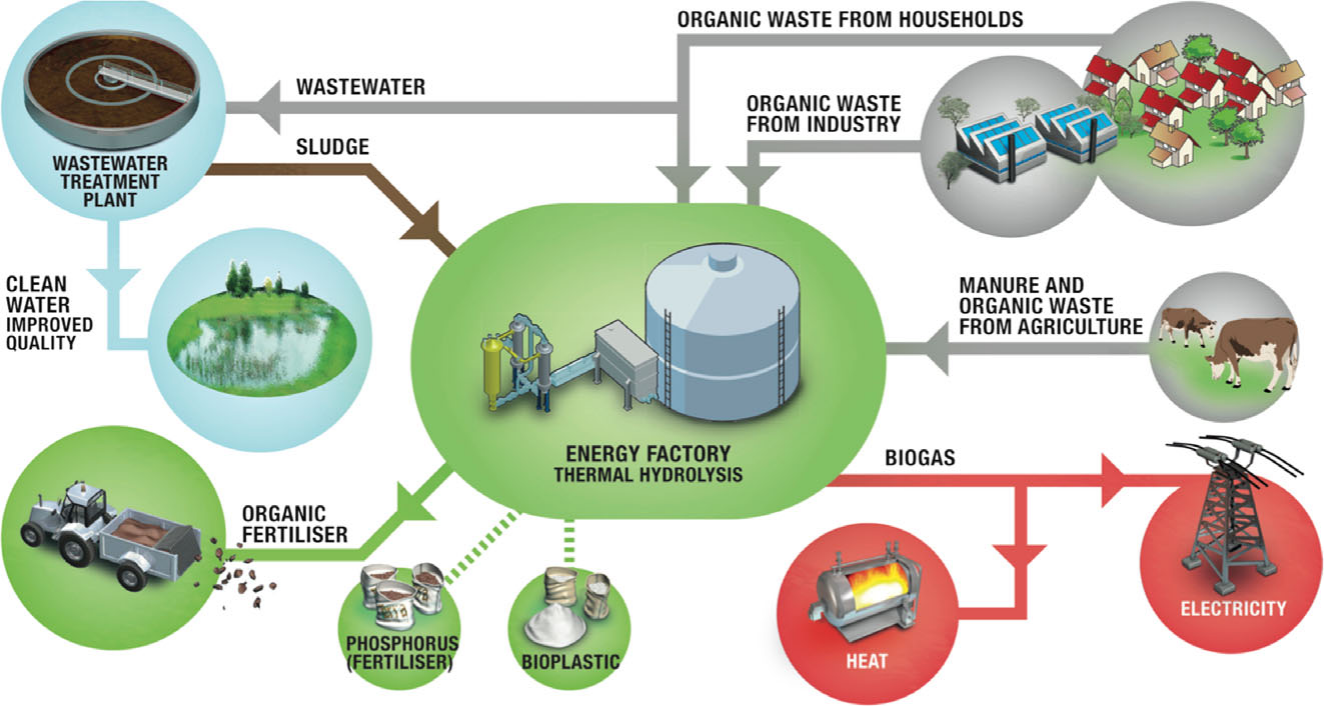
The biorefinery concept in Billund, Denmark. The conventional wastewater treatment plant has been remodelled to carry out the processes indicated, and many other wastewater treatment plants are following with various modifications. See more at http://www.billundbiorefinery.dk/en/.

Introduction of the circular economy approach in wastewater treatment is already being initiated in some countries (Verstraete *et al*., 2007; van Loosdrecht and Brdjanovic, 2014). Typically, wastewater is purified by the activated sludge process, where removal of C, N, P, micropollutants and pathogens takes place. In addition, at many wastewater treatment plants (WWTP), a significant fraction of the incoming wastewater is presettled and added to an anaerobic digester along with surplus sludge from the activated sludge process, all for biogas production and sludge reduction. This can be quite efficient and, e.g. in Denmark, many WWTPs are now energy‐neutral or even net energy producers, sending electricity to the common grid. The energy production can be boosted by adding external waste, e.g. from households, industries or agriculture (Fig. 1). In addition, recovery of phosphorus may also take place, either as struvite from the digester effluent and/or by application of the nutrient‐rich dewatered digester sludge as fertilizer in agriculture (Yuan *et al*., 2012).

Most of the present knowledge about identity and function of specific microbes in wastewater, activated sludge systems and related digesters is summarized in the public online database MiDAS (McIlroy *et al*., 2017). Some microbes involved in the key processes related to nitrification, denitrification, anammox, enhanced biological P‐removal (EBPR) and other processes are now fairly well understood, but there is still a strong need for further studies (see below). Also, when more detailed studies are conducted, processes that were believed to be well‐understood need revision after novel discoveries, such as the discovery of the comammox organisms that oxidize ammonium completely to nitrate (Daims *et al*., 2015; van Kessel *et al*., 2015).

## Needs and perspectives

Microbial biotechnology will continue to initiate, integrate and optimize circular economy in wastewater systems with clear potential for reduced chemical and energy use, and increased energy production and resource recovery. Activated sludge is the dominant reactor type of choice today, but other types, such as biofilms, granules, membrane bioreactors and others, may in some cases be superior to these traditional systems (Pronk *et al*., 2015). However, the process critical microorganisms are often the same, independent of the reactor type, so comprehensive knowledge about identity, physiology, ecology and population dynamics of viruses, bacteria, archaea and higher organisms will be generic and invaluable. This should include a holistic view on incoming microbes, those growing in the different process tanks (activated sludge, digester, side‐stream anammox tanks, others), and all the internal streams of biomass and liquid.

Wastewater, activated sludge and digesters all seem to have a core community of 100–200 abundant genera (each >approx. 0.1% relative abundance) that make up the majority of the biomass and are commonly present across many plants and in many countries (Saunders *et al*., 2016; McIlroy *et al*., 2017). Interestingly, although we know the identity and function of many of these microbes, most are still very poorly characterized and their function basically unknown, although they must play important, so far unknown roles that need to be revealed for a sound understanding of these systems.

In particular, the communities in digesters at WWTPs are poorly investigated, with a very limited number of surveys and few species being well described (Kirkegaard *et al*., 2017). Many digesters suffer from low gas yield, low stability, foaming events and other operational problems that are likely closely connected to the microbial communities (Ganidi *et al*., 2009), so a better understanding is needed to carry out informed control or manipulations of the digester communities. However, as it is not thousands of different abundant species, it is feasible to establish the knowledge needed in the near future of these few hundred relevant species.

There are several specific research needs. The applicability of any microbiological treatment system strongly depends on the stability of the microbial ecosystem. Poor functional stability may result in process breakdown and poor reliability and performance of the system. Such instability has occasionally been reported in WWTP and digesters, but it is not always known whether it is due to variation in the microbial populations or their function. Besides better understanding about the specific species, more general principles governing the stability of such microbial ecosystems should be developed and founded on proper theories in microbial ecology – thus providing a more generic and comprehensive approach to establish and control communities (Curtis *et al*., 2003). Among other specific research needs is a better understanding of the N‐removal with respect to emission of the greenhouse gas N_2_O (Kampschreur *et al*., 2009; Campos *et al*., 2016). It is still unclear which organisms are producing this gas in different systems and how this can be controlled. Another concern in relation to wastewater treatment is the possible dissemination of antibiotic resistance genes into the environment from wastewater treatment plants. The plants are seen as hot spots for transfer of genes (Singer *et al*., 2016), but it is debated how serious a threat this is (Munck *et al*., 2015).

In addition, as it is essential to carry out EBPR (and not apply chemicals) for P recovery through struvite formation or via sludge distribution to the farmland, a better understanding of this process is needed. Several microbes involved in EBPR are now described, but it is still uncertain which species are important in full‐scale plants (Stokholm‐Bjerregaard *et al*., 2017), and there may still be several unrecognized species. Likewise, a serious problem in the daily operation worldwide, poor settling, foam formation or membrane fouling, all due to overgrowth of filamentous microorganisms (Nielsen *et al*., 2009), is only partially resolved despite many years of research. Several filamentous species are now identified and characterized with effective control measures known (Seviour and Nielsen, 2010), but many lack basic characterization, and no control strategies exist.

Newly discovered processes, such as the denitrifying anaerobic methane oxidation (DAMO) process, in which methane is oxidized anaerobically (Raghoebarsing *et al*., 2006; Haroon *et al*., 2013; Cai *et al*., 2015), or novel combinations of processes, such as the integrated sulfate reduction, autotrophic denitrification and nitrification (SANI) process for saline wastewater treatment (Wang *et al*., 2009) also open novel possibilities for developing more sustainable wastewater treatment processes. Many more processes unknown today may contribute to an optimized circular economy in wastewater systems in the future.

Finding alternatives for biogas production that will yield higher levels in the value chain is also a priority. Production of biogas is presently the method of choice at many WWTPs, but the increased need for various biochemical or other high‐value products, such as biohydrogen production (Kleerebezem and van Loosdrecht, 2007) or value‐added chemicals, such as alcohols, organic acids and lipids, which are used as building blocks in the chemical industry or for the synthesis of bioplastics/biopolymers (Kleerebezem *et al*., 2015; Fernandez *et al*., 2015), should be considered. More fundamental changes in the perspectives of recovery of nutrients and organics have also been suggested, including production of proteins (Verstraete *et al*., 2016).

The recent development in DNA sequencing technologies, meta‐omics and other methods provides exciting new possibilities. Within a few years, it will be possible to obtain complete genomes of all important microbes in the systems, including eukaryotes and learn about their physiology and ecology, leading to the next level of ecosystem understanding (Raes and Bork, 2008, Gilbert and Dupont, 2011). Thus, it will be made possible to find selective principles for control of certain populations and management of the communities. Furthermore, ‘online’ surveillance and possibly control of the microbial communities will be possible. Novel DNA sequencing technologies such as Oxford Nanopore MinION can soon be used on‐site for the identification of the complete community within minutes or hours. How to apply such data for surveillance and informed control of the engineered systems is still uncertain, but with an increased research effort across the world, a build‐up of experience and a much better understanding of the microbes’ functions will be gathered and made accessible to the community for practical use, e.g. through MiDAS (McIlroy *et al*., 2017) or other open resources.

In conclusion, wastewater treatment is becoming part of the circular sustainability development and integrates energy production and resource recovery into the production of clean water. Microbial biotechnology is, along with other technologies, essential in this development with exciting perspectives in optimization existing systems and development of new.

## Conflict of interest

None declared.
